# Arterial Tortuosity Syndrome: homozygosity for two novel and one recurrent *SLC2A10* missense mutations in three families with severe cardiopulmonary complications in infancy and a literature review

**DOI:** 10.1186/s12881-014-0122-5

**Published:** 2014-11-06

**Authors:** Marco Ritelli, Nicola Chiarelli, Chiara Dordoni, Elena Reffo, Marina Venturini, Stefano Quinzani, Matteo Della Monica, Gioacchino Scarano, Giuseppe Santoro, Maria Giovanna Russo, Piergiacomo Calzavara-Pinton, Ornella Milanesi, Marina Colombi

**Affiliations:** Division of Biology and Genetics, Department of Molecular and Translational Medicine, School of Medicine, University of Brescia, Viale Europa 11, 25123 Brescia, Italy; Pediatric Cardiology, Department of Pediatrics, University of Padova, School of Medicine, Padova, Italy; Division of Dermatology, Department of Clinical and Experimental Sciences, Spedali Civili University Hospital, Brescia, Italy; Unità Operativa di Genetica Medica, Ospedale Gaetano Rummo, Benevento, Italy; Pediatric Cardiology, A.O.R.N. Ospedale dei Colli, II University of Naples, Naples, Italy

**Keywords:** Arterial Tortuosity Syndrome, *SLC2A10*, Homozygous mutations, Pulmonary artery stenosis

## Abstract

**Background:**

Arterial Tortuosity Syndrome (ATS) is a very rare autosomal recessive connective tissue disorder (CTD) characterized by tortuosity and elongation of the large- and medium-sized arteries and a propensity for aneurysm formation and vascular dissection. During infancy, children frequently present the involvement of the pulmonary arteries (elongation, tortuosity, stenosis) with dyspnea and cyanosis. Other CTD signs of ATS are dysmorphisms, abdominal hernias, joint hypermobility, skeletal abnormalities, and keratoconus. ATS is typically described as a severe disease with high rate of mortality due to major cardiovascular malformations. ATS is caused by mutations in the *SLC2A10* gene, which encodes the facilitative glucose transporter 10 (GLUT10). Approximately 100 ATS patients have been described, and 21 causal mutations have been identified in the *SLC2A10* gene.

**Case presentation:**

We describe the clinical findings and molecular characterization of three new ATS families, which provide insight into the clinical phenotype of the disorder; furthermore, we expand the allelic repertoire of *SLC2A10* by identifying two novel mutations. We also review the ATS patients characterized by our group and compare their clinical findings with previous data.

**Conclusions:**

Our data confirm that the cardiovascular prognosis in ATS is less severe than previously reported and that the first years of life are the most critical for possible life-threatening events. Molecular diagnosis is mandatory to distinguish ATS from other CTDs and to define targeted clinical follow-up and timely cardiovascular surgical or interventional treatment, when needed.

**Electronic supplementary material:**

The online version of this article (doi:10.1186/s12881-014-0122-5) contains supplementary material, which is available to authorized users.

## Background

Arterial Tortuosity Syndrome (ATS; MIM #208050) is a very rare autosomal recessive connective tissue disorder (CTD) characterized mainly by tortuosity and elongation of the large and medium-sized arteries and a propensity for aneurysm formation and vascular dissection. Disease onset usually occurs in infancy or early childhood [1-6]. Additional cardiovascular features include the aberrant origin of aortic branches, pulmonary valve and pulmonary arteries stenosis, and vasomotor instability. ATS also has additional signs that are shared with other CTDs, i.e., Marfan syndrome (MFS), Loeys-Dietz syndrome (LDS), Cutis laxa (CL) and Ehlers-Danlos syndromes (EDS), including soft/velvety/hyperextensible skin, cutis laxa, mildly dysmorphic facial features (i.e., elongated face, hypertelorism, cleft palate and/or bifid uvula, and micro/retrognathia), abdominal hernias, joint hypermobility, congenital contractures and other skeletal anomalies [2-9]. Several patients died at a young age owing mainly to cardiopulmonary complications such as acute respiratory insufficiency and right ventricular (RV) hypertension and hypertrophy, resulting in congestive heart failure, myocarditis and ischemic events leading to organ infarction [3]. Stenosis of the pulmonary arteries (PAS) occurs in 60% of the patients [5], and in severe cases, there is significant obstruction to RV output, causing RV hypertension and dysfunction. All ATS patients require regular follow-up and may benefit from surgical interventions, i.e., aortic root replacement for aortic aneurysms; pulmonary artery reconstruction has yielded good clinical and hemodynamic outcomes in specific patients [5,10-15].

ATS is caused by mutations in the *SLC2A10* gene encoding the 541-amino-acid facilitative glucose transporter 10 (GLUT10) [4]. GLUT10 belongs to the SLC2A transporter family and facilitates the uptake of D-glucose, D-galactose and 2-deoxy-D-glucose, and dehydroascorbic acid (DHA) [16-18]. Here, we report the clinical findings and molecular characterization of three novel ATS families with acute respiratory symptoms in infancy and describe two novel *SLC2A10* mutations. We compare the clinical features of the patients with previous data to elucidate the clinical phenotype and natural history of the disorder.

## Case presentation

### Patients

Patients were evaluated by pediatric cardiologists, clinical geneticists and dermatologists. Written informed consent was obtained for each proband and family member before sample collection for genetic analyses. Specific consent was obtained for publication of the clinical data including photographic documentation. This study was approved by the medical ethics committee of the University Hospital Spedali Civili of Brescia and was performed in accordance with the Declaration of Helsinki Principles.

Although the three evaluated ATS families lacked consanguinity, the parents of three patients had a common geographical origin. In the first family, the proband was a 14-year-old Italian boy, his older brother was unaffected. In the second family, the probands were two Macedonian 8- and 6-year-old brothers born after an elder healthy brother. In the third Italian family, the patient clinically diagnosed with ATS died at 26 months of age during cardiovascular surgery; his healthy parents were characterized by *SLC2A10* genetic testing. The main clinical features of the four patients are shown in Table 1.

**Table 1:** **Summary of the main clinical features of the ATS patients described in this study**

	**Patient 1**	**Patient 2**	**Patient 3**	**Patient 4**
	**Italian (M/14)**	**Macedonian (M/8)**	**Macedonian (M/6)**	**Italian (M/died at 26 months of age)**
**Age of onset/presenting symptom(s)**	Birth/cyanosis, congenital RV hypertension due to PAS	1 month/cyanosis	3 months/respiratory failure and cyanosis due to PAS	First months/cyanosis after feeding
***Dysmorphisms***				
**Elongated face**	+	+	+	
**Scaphocephaly (mild)**	+	-	-	
**Beaked nose**	+	+	+	
**Micro-retrognathia**	+	+	+	
**High-arched palate**	-	+	+	
**Uvula abnormalities**	+	+ (bifid)	+ (elongated)	
***Skin***				
**Doughy**	+	+	+	
**Hyperextensible**	+	-	-	
**Atrophic scars**	+	+	-	
***Cardiovascular***				
**Tortuosity**	PA, Brachiocephalic vessels	Intraparenchymal PA, aortic arch, ascending aorta, brachiocephalic vessels	Intraparenchymal PA, aortic thoracic tract	PA, supra-aortic vessels, aortic arch
**Pulmonary arteries defects**	Stenotic peripheral PA	Marked tortuosity of intraparenchymal PA, stenosis of pulmonary branches	Tortuosity of intraparenchymal PA, dilatation of main PA and central branches	Tortuosity of PA, dilatation of pulmonary trunk
**RV hypertension**	+ Birth	-	+ 50 mmHg	+ 110 mmHg
**RV anomalies**	+	+ Dilatation	+ Hypertrophy, left ventricle compression	+ Dilatation
**Valvular regurgitation**	Mitralic, mild	Tricuspidal, mild	Tricuspidal, mild	Tricuspidal, mild
**Surgery**	Reconstructive surgery of pulmonary vessels at 1 years; stenting of PA at 2 years	-	-	Reductive pulmonary arterioplasty with stenting of PA branches
**Easy bruising**	+	+	+	
***Other***				
**Hypertrophic pyloric stenosis**	+	-	-	-
**Keratoconus**	+	-	-	
**Joint hypermobility (Beighton score)**	-	+ (7/9)	+ (5/9)	

## Methods

### Mutation screening

Genomic DNA was extracted from peripheral blood leukocytes using standard procedures; the exons and intron-flanking regions of the *SLC2A10* gene were PCR amplified and directly sequenced using an ABI3130XL Genetic Analyzer. Segregation was verified in all families; the causal mutations were absent in a panel of 200 healthy donors. The nucleotide and protein accession numbers correspond to the *SLC2A10* (NM_030777.3; NP_110404.1) reference sequence. Mutations were annotated according to the HGVS nomenclature (www.hgvs.org/mutnomen). Nucleotide numbering is based on the cDNA sequence numbering, with +1 corresponding to the A of the ATG translation initiation codon 1 in the reference sequence. For protein numbering, +1 corresponds to the first translated amino acid. We used the mutation-prediction programs MutationTaster (www.mutationtaster.org), PolyPhen-2 (http://genetics.bwh.harvard.edu/pph2), project HOPE (http://sift.jcvi.org/), SIFT (www.sift.jcvi.org) and Alamut version 2.2 to evaluate causality based on alteration of the protein structure.

## Results

### Clinical findings

**Figure 1:**
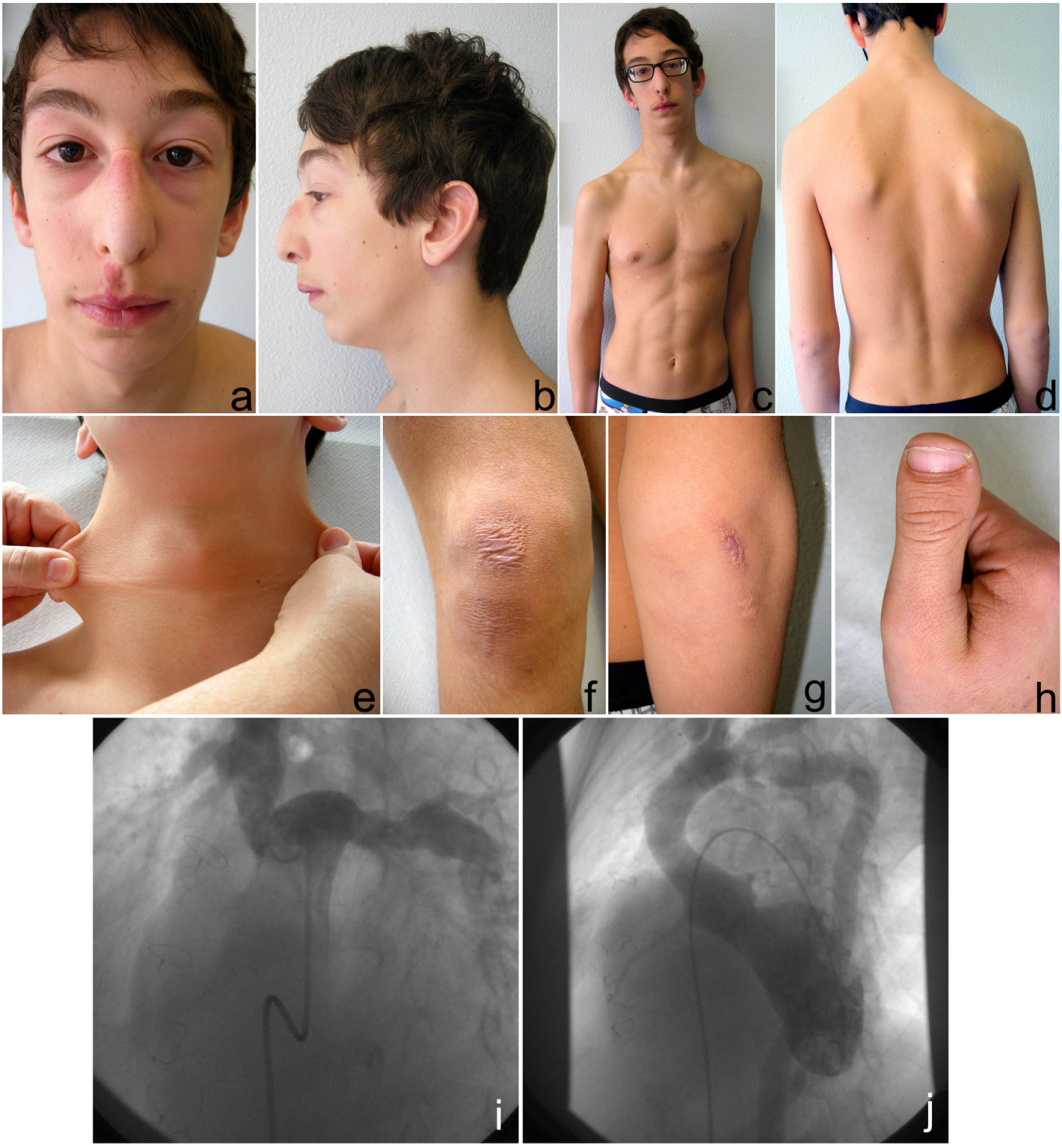
**Clinical and angiographic features of Patient 1:** mild scaphocephaly, elongated face, beaked nose, microretrognathia, elongated philtrum with congenital angioma, large ears **(a,b)**, winged scapulae, scoliosis **(c,d)**, hyperextensible skin over the neck **(e)**, atrophic scar over the right the knee and elbow **(f,g)**, and brachydactyly type D **(h)**. An angiographic study performed between the pulmonary vessel reconstructive surgery and the following stenting revealed the tortuosity of the aorta and the origin of the brachiocephalic vessels **(i)** and the narrow origin of the pulmonary artery branches and their tortuous course **(j)**.

**Patient 1** (Family A). The 14-year-old Italian proband was born following an uneventful pregnancy and delivery. The weight at birth was 4.2 kg, which was in the 50^th^-75^th^ centile. He presented with cyanosis spontaneously resolved (Table 1). His perinatal period and psychomotor development were normal. The patient’s hypertrophic pyloric stenosis was surgically corrected at 40 days of age. Severe RV hypertension due to stenotic and tortuous pulmonary artery branches was resolved by surgical arterioplasty at 1 year of age (Figure 1, i) and with stenting of pulmonary arteries at 8 years. Aortography performed during the interventional cardiac catheterization revealed an elongated and tortuous aorta and tortuous brachiocephalic vessels (Figure 1, j) as well as mild mitral valve regurgitation. Keratoconus and myopia were diagnosed at 5 years.

The following features were observed on examination at 14 years of age: elongated face, beaked nose, large ears, microretrognathia, elongated philtrum, congenital angioma over the philtrum, high-arched palate, doughy skin, atrophic scars over the right knee and the elbows, striae distensae over the glutei, brachydactyly type D, patella hyperlaxity, absence of joint hypermobility according to the Beighton score (0/9), leg-length discrepancy, winged scapulae, scoliosis, and genua valga (Figure 1, a-h).

**Figure 2:**
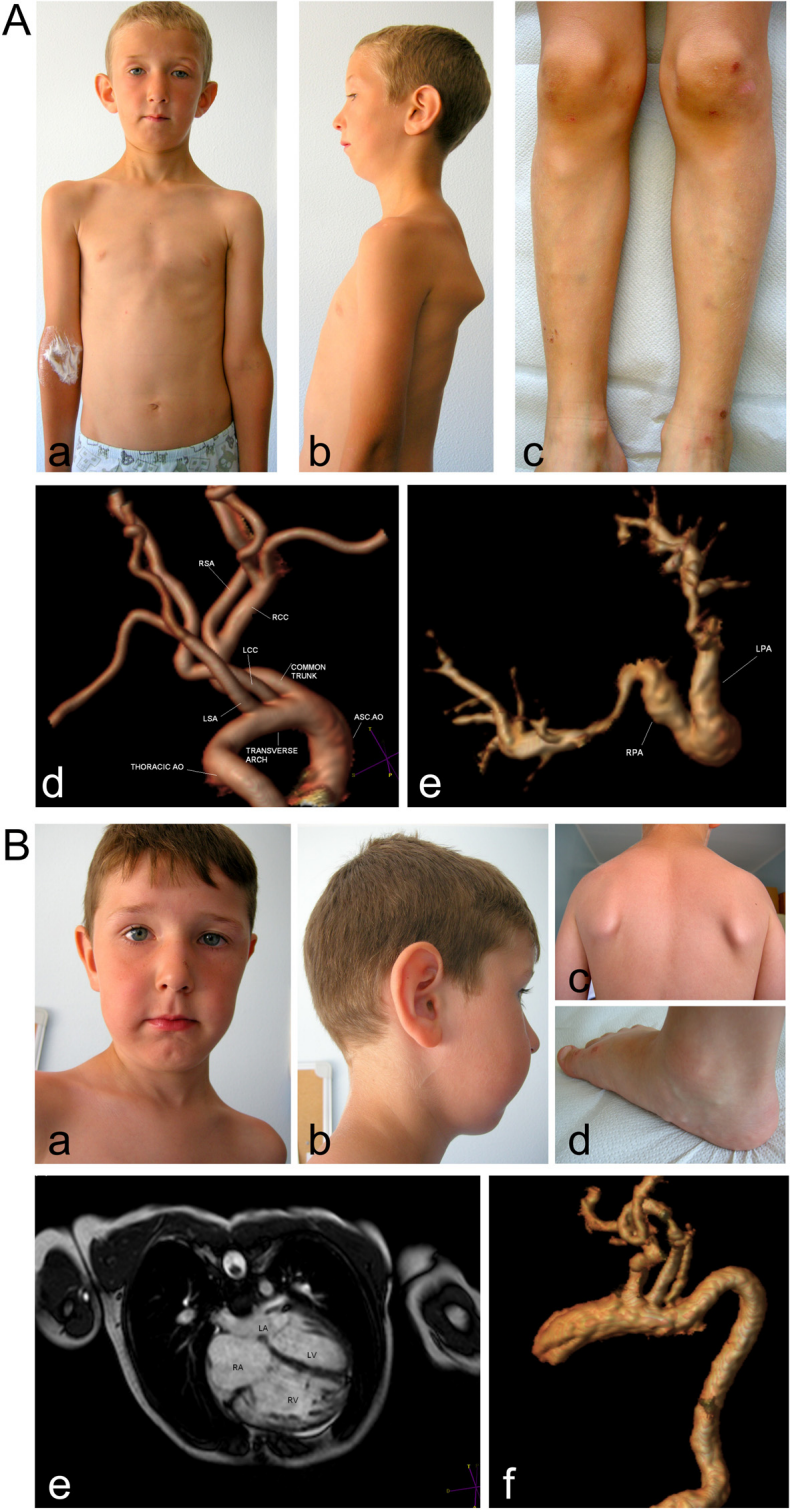
**Clinical and radiological features of Patients 2 and 3. A)** Patient 2: mild scaphocephaly, hypotelorism, large ears, elongated face, micro-retrognathia, winged scapulae, scoliosis **(a,b)**, genua valga, easy bruising, small atrophic scar over left knee **(c)**, tortuosity of aortic arch and brachiocephalic vessels **(d)**, marked tortuosity of intraparenchymal arteries **(e)**. **B)** Patient 3: mild scaphocephaly, epicanthus, hypotelorism, large ears, elongated face, microretrognathia **(a,b)**, winged scapulae, scoliosis **(c)**, pes planus, piezogenic papules **(d)**, right ventricular hypertrophy with left ventricular compression **(e)**, marked tortuosity of intraparenchymal arteries **(f)**.

**Patient 2** (Family B). The 8-year-old Macedonian boy was born following an uneventful pregnancy and delivery. His psychomotor development was normal. At 1 month of age, he presented with severe respiratory distress and cyanosis (Table 1). Easy bruising, dyspnea after physical exercise, and chronic fatigue syndrome were observed throughout his childhood. A heart ultrasound performed at 8 years of age revealed mild RV dilatation and mild tricuspidal regurgitation. A heart magnetic resonance angiography (MRA) revealed an irregular ascending aorta, marked tortuosity of the intraparenchymal pulmonary arteries, and PAS (Figure 2A, d,e). The systolic pulmonary artery pressure was normal.

On examination at 8 years, the patient’s height was 1.23 m (10^th^-25^th^ centile), and his weight was 22 kg (10^th^-25^th^ centile). The dysmorphic features were large and protruding ears, elongated face, microretrognathia, (Figure 2A, a,b), epicanthal folds, blepharophimosis, high-arched palate, and elongated uvula with median raphe. The skin was doughy and poorly hyperextensible; small atrophic scars were present over the left knee, and ecchymoses were observed in the pretibial region (Figure 2A, c). The skeletal signs included joint hypermobility according to the Beighton score (7/9), mild pectus excavatum, scoliosis, winged scapulae (Figure 2A, b), patella hyperlaxity, genua valga, and pedes plani.

**Patient 3** (Family B). The 6-year-old Macedonian patient was born following an uneventful pregnancy and delivery. His psychomotor development was normal. At 3 months of age, he presented with respiratory failure and cyanosis secondary to peripheral PAS; severe dyspnea and cyanosis recurred in his childhood after moderate physical exercise, and easy bruising and chronic fatigue syndrome also occurred (Table 1). A heart ultrasound revealed mild tricuspidal regurgitation, RV hypertrophy and left ventricular compression. At 6 years of age, a heart MRA revealed dilatation of the main pulmonary artery and central branches, tortuosity and stenosis of the intraparenchymal pulmonary arteries and high systolic pressure in the central pulmonary arteries (50 mmHg, n.v. 15–30) (Figure 2B, e). Furthermore, the aortic thoracic tract had a tortuous appearance (Figure 2B, f).

On examination at 6 years of age, his height was 1.15 m (25^th^-50^th^ centile) and weight was 20 kg (25^th^ -50^th^ centile). The observed dysmorphisms included large ears, elongated face, microretrognathia, high palate (Figure 2B, a,b), and elongated uvula; the skin was doughy and poorly hyperextensible; small atrophic scars were present over the knees, and ecchymoses were observed in the pretibial region; piezogenic papules were present on the heels (Figure 2B, d). The skeletal signs included joint hypermobility according to the Beighton score (5/9), mild pectus excavatum, winged scapulae, scoliosis (Figure 2B, c), patella hyperlaxity, genua valga, and pedes plani.

**Patient 4** (Family C). The patient was brought in for medical attention in the first months of life because of cyanosis after feeding, and in-depth vascular studies were performed (Table 1). At 4 months of age, aortography and pulmonography revealed tortuosity of the supra-aortic vessels, aortic arch, and pulmonary arteries, which also showed severe stenosis. A heart ultrasound performed at 20 months of age revealed left ventricular hypertrophy and severe pulmonary hypertension (110 mmHg) with RV and pulmonary trunk dilatation. A clinical diagnosis of ATS was made. At 26 months of age, the child was admitted for cardiovascular surgery, and reductive pulmonary arterioplasty with stenting of the pulmonary artery branches was performed. An intraoperative examination revealed marked dilatation of the pulmonary trunk, which was three times larger than the ascending aorta. When the patient was weaned from extracorporeal circulation, the patient died as a result of secondary decompensation leading to impaired cardiac contractile function and subsequent cardiac arrest.

### Molecular analyses

**Figure 3:**
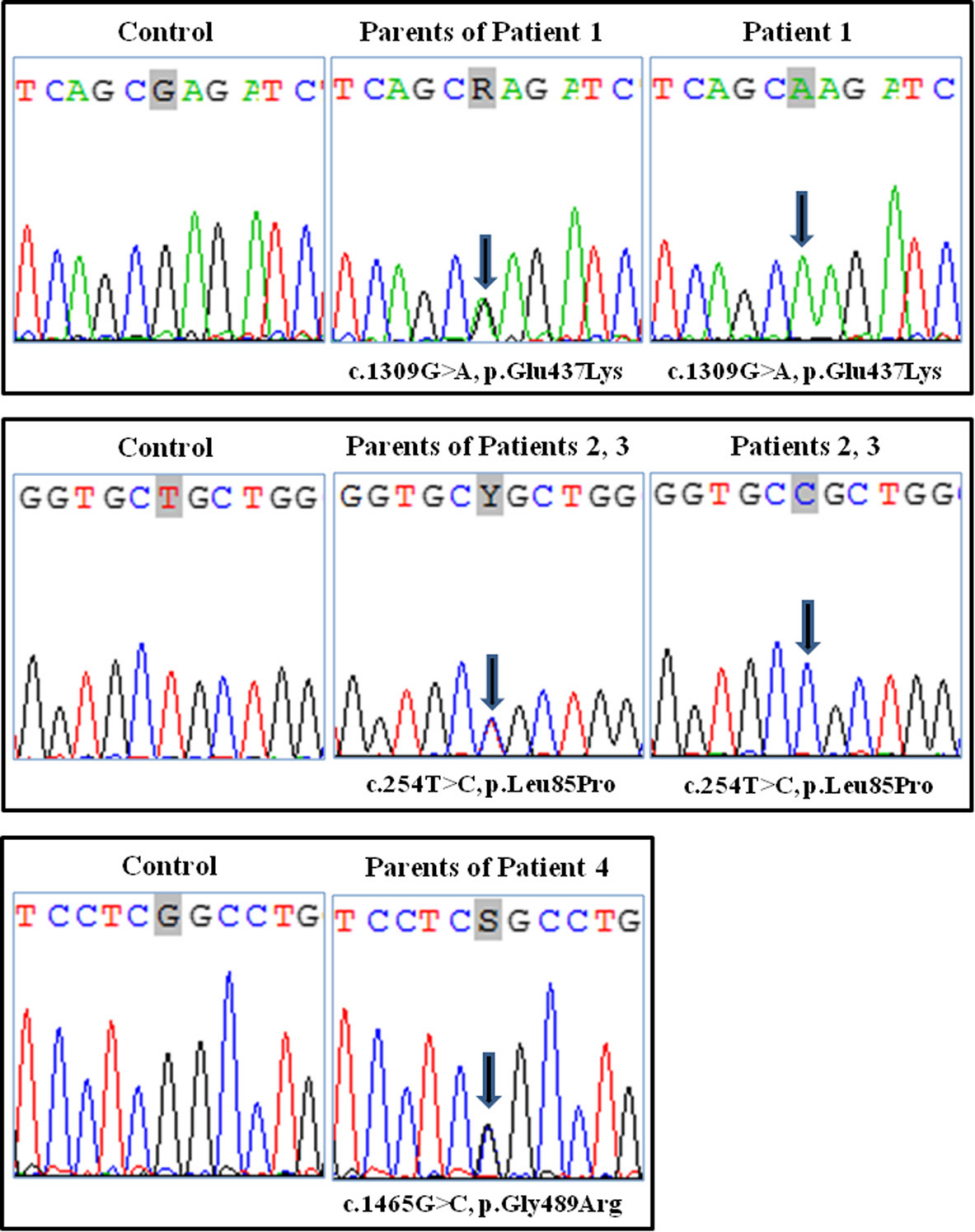
**Molecular characterization.** Sequencing of *SLC2A10* exons and splice junctions revealed the following: Patient 1 was homozygous for the recurrent mutation c.1309G>A (p.Glu437Lys) in exon 3; Patients 2 and 3 were homozygous for the novel mutation c.254T>C (p.Leu85Pro) in exon 2; the parents of Patient 4 were heterozygous for the novel mutation c.1465G>C (p.Gly489Arg) in exon 4.

In Patient 1 (Family A), sequencing analysis revealed the homozygous c.1309G>A transition in exon 3 of the *SLC2A10* gene; both parents of the proband were heterozygous for this mutation (Figure 3). This causal mutation was previously identified in compound heterozygosis in another Italian patient and substitutes a glutamic acid residue with a basic lysine residue (p.Glu437Lys) at the second position of the 5^th^ intracellular GLUT10 loop; the glutamic acid residue at this position is highly conserved in all members of the SLC2A glucose transporter gene family and in *SLC2A10* orthologs in other species [8].

In Patients 2 and 3 (Family B), *SLC2A10* genotyping revealed the homozygous novel c.254T>C transition in exon 2, resulting in the p.Leu85Pro missense mutation; both parents of these probands were heterozygous for this mutation (Figure 3). In silico prediction algorithms (SIFT score 0.01, MutationTaster p value 1.000, PolyPhen2 HumDiv score 0.999, HumVar score 0.977, and Grantham score 98) characterized the p.Leu85Pro substitution as deleterious and probably damaging. This mutated and highly conserved residue is located in the 3^th^ transmembrane domain (TMD3) and is located near the recurrent p.Ser81Arg missense mutation [4,19].

During prenatal genetic counseling in Family C, genetic testing revealed that the parents of Patient 4 were heterozygous carriers of the novel mutation c.1465G>C (Figure 3). This transversion occurs in exon 4 of the *SLC2A10* gene, substituting a neutral glycine residue with a positively charged arginine residue (p.Gly489Arg) in the last transmembrane domain (TMD12) of the GLUT10 protein. The in silico prediction algorithms (SIFT score 0.01, MutationTaster p value 0.999, PolyPhen2 HumDiv score 0.976, HumVar score 0.898, and Grantham score 125) characterized the p.Gly489Arg substitution as deleterious and probably/possibly damaging. This molecular characterization allowed the prenatal analysis of a chorionic villus sample in the second pregnancy, which revealed that the fetus was heterozygous for the familial mutation.

## Discussion

**Figure 4:**
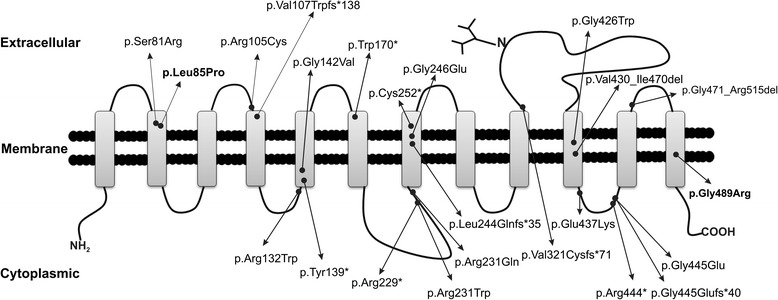
**Graphical overview of all known** ***SLC2A10*** **mutations.** GLUT10 contains 12 hydrophobic TMDs (rectangles), a hydrophilic endofacial loop between TMDs 6 and 7 as well as a large hydrophilic loop containing a putative N-linked glycosylation site between TMDs 9 and 10. The two novel mutations identified in this study are indicated in bold letters. The numbering is based on the protein reference sequence NP_110404.1.

ATS, first described by Ertrugul in 1967 [20] and Beuren et al., in 1969 [21], is a very rare CTD caused by recessive *SLC2A10* mutations. Approximately 100 ATS patients have been reported, including those from inbred multiplex families in Italy, Morocco, Saudi Arabia, and Qatar [4,7,19,22,23]. The majority of the reported ATS patients are infants or children [2,5,9,23,24]. A total of 23 mutations have been identified in the *SLC2A10* gene: 12 missense substitutions, 5 nonsense nucleotide changes, 5 deletions leading to frame-shifts and premature termination codon formation and one splice mutation ([4-9,19,23,25-27], this study) (Figure 4 and Additional file 1: Table S1). Although the pathogenic mechanism underlying all the *SLC2A10* mutations is the loss of GLUT10 function, the specific role of the GLUT10 transporter in ATS pathogenesis is still controversial. Similar to other conditions known as TGF-β vasculopathies (MFS, which is caused by *FBN1* mutations and LDS, which is caused by *TGFBR1*, *TGFBR2*, *SMAD3* and *TGFB2* mutations), the *SLC2A10* loss of function activates the TGF-β signaling pathway [4,28]. Considering that GLUT10 is a putative DHA transporter, diminished GLUT10 activity in ATS might lead to structural abnormalities in the arterial wall proteins such as collagens and elastin due to a reduction in hydroxylated prolines and lysines or to oxidative stress injury caused by elevated production of reactive oxidative species. These abnormalities would result in pathogenic vascular remodeling [17,18]. However, in both the “endoplasmic reticulum – enzyme cofactor” and “mitochondrial – antioxidant” models of vitamin C-related pathology, the exact interaction with the TGF-β pathway in ATS remains unclear and requires further study [29,30].

The clinical findings of the ATS patients described in this study and the families previously characterized by our group are summarized in Additional file 2: Table S2 [2,4,6,8-12,31]. These patients constitute a unique cohort of clinically and molecularly defined Italian ATS patients, with the exception of the two Macedonian brothers described here. Our cohort comprised 12 patients from 8 different families; consanguineous relationships were present in two of these families. Apart from arterial tortuosity, that is present in all patients of our cohort, the signature features of ATS seem to be a facies suis generis including a long face, malar hypoplasia, a beaked nose, and micrognathia, as well as a high frequency of PAS observed in 75% of the patients (9/12). In addition to these hallmarks, ATS has several somatic features shared with other CTDs; therefore, clinical diagnosis can require further examination by echocardiography (ECG), angiography, and MRA and/or CT scan. The somatic signs of CTDs in our cohort comprised easy bruising (9/9), doughy skin (9/11) with generally mild hyperextensibility (4/10), joint hypermobility (9/11), relapsing hernias (7/12), valvular regurgitation (8/10), and keratoconus (3/8). Aortic aneurysms might not be a signature feature of ATS because aneurysms were observed only in 3 patients, and dissections did not occur. A small aneurysm of the splenic artery was observed in the patient of Family D at 51 years of age, and an ascending and aortic root aneurysm was observed in 2 members of Family H, respectively. The mild splenic artery aneurysm and the ascending aneurysms did not require surgery, whereas a valve-sparing aortic root replacement was performed to prevent further aortic dilation and rupture in the patient of family H at 19 years of age [11].

In our cohort, the mean age at clinical examination was approximately 15 years. Eleven patients, 7 males and 4 females, are still alive with a mean age of approximately 23 years. One patient died at 26 months of age following cardiovascular surgery for PAS. Four of the surviving patients are children with a mean age of approximately 9 years, and seven are adults with a mean age of approximately 31 years, including a 55-year-old patient. Therefore, consistent with the study by Callewaert et al., [5], in our cohort, the most striking observation is that the life expectancy of patients with ATS is longer than initially observed [3]. Wessels et al., [3] observed that ATS is fatal before the age of 5 years in 40% of patients. This conclusion was based on a review of 35 ATS patients; 26 of these patients were observed previously and had a high rate of consanguinity [1,10,20,21,32-37]; the other 9 patients were novel cases from 3 other consanguineous families. Subsequently, Callewaert et al., [5] described 16 new patients with defined *SLC2A10* mutations and with a low rate of consanguinity (25%); the mean age of these patients (13 years) was greater than previous observations, and only one patient died at 33 years of age, most likely from an unrelated cause. One potential reason for the initial bias towards low life-expectancy/high mortality rate might be the lack of molecular confirmation in the patients studied by Wessel et al., [3]; these patients were studied before the casual gene was identified [4]. Molecular testing is often mandatory to distinguish ATS from other CTDs with strictly overlapping clinical signs and with high rates of life-threatening cardiovascular events such as LDS or autosomal recessive CL [38,39]. Furthermore, the high rate of consanguinity in the families studied by Wessel et al., [3] might have worsened the phenotype. Finally, in recent years, increased knowledge on the disease has facilitated targeted follow-ups and timely cardiovascular surgery when needed, leading to improved prognosis. Therefore, the first few years of life might be critical for potentially life-threatening events [26,27,40]. Callewaert et al., [5] observed that 4 out of 16 patients had cyanosis in the first few months of life, and one patient experienced a stroke at 8 months of age. Furthermore, in our cohort, the first clinical presentation, i.e., acute respiratory symptoms such as cyanosis or respiratory distress occurred in the majority of the cases (50%) at birth or during the first few months of life. All the new patients described in this study presented with severe cardiopulmonary complications in infancy, and in one case, these complications were fatal. Because PAS is the most dangerous cardiovascular feature in ATS patients, Ekici et al., [15] proposed that the clinical outcome was directly related with the degree of PAS. Al-Khaldi et al., [14] described 7 pediatric patients with bilateral peripheral and central PAS with systemic to supra-systemic RV pressure and severe RV dysfunction. All these patients underwent successful surgical repair for reconstruction of the pulmonary arterial tree, indicating that this is an excellent and safe strategy for severe ATS patients. In our cohort, combined surgical and interventional treatment allowed Patient 1 (Family A) and patient of Family F [12] to reach adolescence in a good hemodynamic condition without any pharmacologic support with only mildly elevated RV pressure and good RV and LV function and to lead a normal life including all activities appropriate to their age (NYHA class I). The same hybrid surgical procedure was adopted also for Patient 4, unfortunately the patient died during weaning from extracorporeal circulation as a result of secondary decompensation leading to impaired cardiac contractile function. However, to our knowledge [11,12] and based on a literature review [13-15], complications have not been observed during cardiovascular surgery in ATS patients, and the risk of fatal events should be similar to the general population. Together, these data suggest that in ATS, an early diagnosis is life saving, leading to proper management and follow-up. Specific complications that may appear in adulthood include chronic systemic and pulmonary hypertension, heart conduction defects, aortic root dilatation, stroke, and intracranial aneurysms [2,5,9,11,24]. These features that occur later during life necessitate specific attention and management. In addition to the use of vascular imaging (MRA and/or CT scan) as previously described [5], specific additional less-invasive follow-up procedures might include periodic measurement of systemic blood pressure, ECG, and heart ultrasound with aortic root diameter measurements [9].

The obstetric aspects of ATS have not been elucidated. Allen et al., [41] reported a 27-year old ATS woman with successful pregnancy. In our cohort, 3 out of 4 women had pregnancies without complicated deliveries. These data suggest that in ATS, pregnancy can be safely handled with multidisciplinary management including close maternal and fetal surveillance.

## Conclusions

In conclusion, our data confirm that cardiovascular prognosis in ATS is less severe than previously reported and that the first few years of life are the most critical for possible life-threatening events, i.e., acute respiratory symptoms, due mostly to complications of PAS. Molecular diagnosis is mandatory to distinguish ATS from other CTDs with strictly overlapping clinical signs and with a high rate of more severe cardiovascular events and to define targeted clinical follow-up and timely cardiovascular surgery when needed. Further studies on the functional properties of GLUT10 are required to elucidate the pathogenic mechanisms involved in ATS etiology to identify potential therapeutic options.

### Consent

Written informed consent was obtained from patients’ parents for publication of this case report and accompanying images. A copy of the written consent is available for review by the Editor-in-Chief of this journal.

## Additional files

Additional file 1: Table S1. Overview of currently known *SLC2A10* mutations. Additional file 2: Table S2. Summary of the clinical features of the ATS patients described in the present study and previously reported by our group.

## Abbreviations

ATS: Arterial Tortuosity Syndrome
CL: Cutis laxa
CTD: Connective tissue disorder
CT: Computerized tomography
DHA: Dehydroascorbic acid
ECG: Echocardiography
EDS: Ehlers-Danlos Syndrome
LDS: Loeys-Dietz Syndrome
MFS: Marfan Syndrome
MRA: Magnetic resonance angiography
NYHA: New York Heart Association
PAS: Stenosis of the pulmonary arteries
RV: Right ventricular
TMD: Transmembrane domain
