# Supplementary material for: Arterial Tortuosity Syndrome: homozygosity for two novel and one recurrent SLC2A10 missense mutations in three families with severe cardiopulmonary complications in infancy and a literature review
Source: BMC Med Genet. 2014 Nov 6;15:122. doi: 10.1186/s12881-014-0122-5 (PMC4412100; doi:10.1186/s12881-014-0122-5)
Supplement: Additional file 1: Table S1. — Overview of currently known SLC2A10 mutations. [file 12881_2014_122_MOESM1_ESM.doc]

**Table S1.** **Overview of currently known *SLC2A10* mutations**

| ***Exons*** | ***Coding sequence change*** | ***Amino acid change*** | ***Functional domains*** | ***References*** |
| --- | --- | --- | --- | --- |
| **2** | c.243C>G | p.Ser81Arg | TMD3 | [4,19] |
| **2** | **c.254T>C** | **p.Leu85Pro** | **TMD3** | **Novel, this study** |
| **2** | c.313C>T | p.Arg105Cys | Exofacial loop between TMD3 and TMD4 | [23] |
| **2** | c.318del | p.Val107Trpfs*138 | TMD4 | [25] |
| **2** | c.394C>T | p.Arg132Trp | Endofacial loop between TMD4 and TMD5 | [5] |
| **2** | c.417T>A | p.Tyr139* | TMD5 | [27] |
| **2** | c.425G>T | p.Gly142Val | TMD5 | [5] |
| **2** | c.510G>A | p.Trp170* | TMD6 | [4,26] |
| **2** | c.685C>T | p.Arg229* | Endofacial loop between TMD6 and TMD7 | [5,6,41] |
| **2** | c.691C>T | p.Arg231Trp | Endofacial loop between TMD6 and TMD7 | [6] |
| **2** | c.692G>A | p.Arg231Gln | Endofacial loop between TMD6 and TMD7 | [5,27] |
| **2** | c.731_734del | p.Leu244Glnfs*35 | TMD7/Q242QLTG sugar transporter signatures | [5] |
| **2** | c.737G>A | p.Gly246Glu | TMD7/Q242QLTG sugar transporter signatures | [5] |
| **2** | c.756C>A | p.Cys252* | TMD7 | [6,7] |
| **2** | c.961del | p.Val321Cysfs*71 | Exofacial loop between TMD9 and TMD10 | [4] |
| **2** | c.1276G>T | p.Gly426Trp | TMD10 | [5] |
| **3** | c.1309G>A | p.Glu437Lys | Endofacial loop between TMD10 and TMD11 | [8], **this study** |
| **3** | c.1330C>T | p.Arg444* | Endofacial loop between TMD10 and TMD11 | [8] |
| **3** | c.1334delG | p.Gly445Glufs*40 | Endofacial loop between TMD10 and TMD11 | [4,5] |
| **3** | c.1334G>A | p.Gly445Glu | Endofacial loop between TMD10 and TMD11 | [5] |
| **3** | c.1411+1G>A | p.Val430_Ile470del | TMD10-TMD12 | [9] |
| **4** | c.1411+480_c.1547+299del | p.Gly471_Arg515del | Exofacial loop between TMD11 and TMD12, C- term | [5] |
| **4** | **c.1465G>C** | **p.Gly489Arg** | **TMD12** | **Novel, this study** |
