# Supplementary material for: Arterial Tortuosity Syndrome: homozygosity for two novel and one recurrent SLC2A10 missense mutations in three families with severe cardiopulmonary complications in infancy and a literature review
Source: BMC Med Genet. 2014 Nov 6;15:122. doi: 10.1186/s12881-014-0122-5 (PMC4412100; doi:10.1186/s12881-014-0122-5)
Supplement: Additional file 2: Table S2. — Summary of the clinical features of the ATS patients described in the present study and previously reported by our group. [file 12881_2014_122_MOESM2_ESM.doc]

**Supplementary Table 2:** Summary of the clinical features of the ATS patients described in the present study and previously reported by our group.

|  | **Family A^*^** | **Family B^*^** | | **Family C^*^** | **Family D^1^** | **Family E^2,3^** | **Family F ^3,4^** | **Family G^5,6^** | **Family H^7,8,9^** | | | |
| --- | --- | --- | --- | --- | --- | --- | --- | --- | --- | --- | --- | --- |
| Sex/age on  examination | M/14 y | M/8 y | M/6 y | M/26 m | F/51 y | M/ 8 m | M/5 y | M/14 y | M/16 y | F/21 y | F/21 y | F/19 y |
| Present age | 16 | 9 | 7 | dead | 55 | 6 | 10 | 31 | 28 | 31 | 31 | 29 |
| Consanguinity | **-** | **-** | **-** | - | **+** | **-** | **-** | **-** | **+** | **+** | **+** | **+** |
| Age of onset/  presenting symptom(s) | Birth/  cyanosis, congenital RV hypertension | 1 m/ cyanosis | 3 m/  respiratory failure/cyanosis due to PAS | 2 m/  cyanosis | 10 y/  inguinal hernias | Birth/  respiratory distress | 4 y/  dyspnea | 5 y/  persistent hypotonia | Delivery/ cardiac arrest | Birth/  inguinal hernias | Childhood/dyspnea | Childhood/dyspnea |
| **DYSMORPHISMS** | | | | | | | | | | | | |
| Aged appearance | **-** | **-** | **-** |  | **+** | **-** | **-** | **-** | **-** | **-** | **-** | **-** |
| Long face | **+** | **+** | **+** |  | **+** | **+** | **+** | **+** | **+** | **+** | **+** | **+** |
| Beaked nose | **+** | **-** | **-** |  | **+** | **+** | **-** | **+** | **+** | **+** | **+** | **+** |
| Down palpebral slant | **+** | **+** | **-** |  | **-** | **+** | **+** |  | **+** | **+** |  |  |
| Blepharophimosis | **-** | **-** | **-** |  | **+** | **+** | **-** | **-** | **-** | **-** | **-** | **-** |
| Malar hypoplasia | **+** | **+** | **-** |  | **+** | **+** | **-** | **+** | **+** | **+** | **+** | **+** |
| Uvula abnormalities | **-** | **+** | **+** |  | **-** |  | **-** |  |  |  |  |  |
| High-arched palate | **+** | **+** | **+** |  | **-** |  | **-** |  |  |  |  |  |
| Microretrognathia | **+** | **+** | **+** |  | **-** | **+** | **-** | **+** | **+** | **+** | **+** | **+** |
| **OCULAR** | | | | | | | | | | | | |
| Keratoconus | **+** |  |  |  | **-** |  | **-** | **+** | **-** | **+** | **-** | **-** |
| Corneal dystrophy | **+** | **-** | **-** |  | **+** | **-** | **-** | **-** | **-** | **-** | **-** | **-** |
| Myopia | **+** | **-** | **-** |  | **+** |  | **-** | **-** | **-** | **-** | **+** | **-** |
| **SKIN** | | | | | | | | | | | | |
| Doughy | + | + | + |  | + | - | - | + | + | + | + | + |
| Hyperextensible | + | + | + |  | - | - | - |  | - | + | - | - |
| Hernia(s) | + i | - | - | + i | + i, u, l | + s | - | + i | + i | + i | - | - |
| Defective scars | + |  | - |  | + | - | - |  | - | - | - | - |
| **SKELETAL** | | | | | | | | | | | | |
| Pectus excavatum | **-** | **+** | **+** |  | **-** | **-** | **+** | **-** | **-** | **-** | **-** | **-** |
| Scoliosis | **+** | **+** | **+** |  | **-** | **-** | **+** | **+** | **-** | **+** | **-** | **-** |
| Winged scapulae | **+** | **+** | **+** |  | **-** | **-** | **-** |  | **-** | **-** | **-** | **-** |
| Genua valga | **+** | **+** |  |  | **+** | **-** | **-** | **+** | **-** | **-** | **-** | **-** |
| Pedes plani/cavi | **-** | **+** | **+** |  | **-** | **-** | **-** |  | **-** | **-** | **-** | **-** |
| Joint hypermobility | **-** | **+** | **+** |  | **+** | **-** | **+** | **+** | **+** | **+** | **+** | **+** |
| Chronic pain | **-** | **-** | **-** |  | **+** | **-** | **-** | **-** | **-** | **-** | **-** | **-** |
|  | **Family A^*^** | **Family B^*^** | | **Family C^*^** | **Family D^1^** | **Family E^2,3^** | **Family F ^3,4^** | **Family G^5,6^** | **Family H^7,8,9^** | | | |
| Sex/age on  examination | M/14 y | M/8 y | M/6 y | M/26 m | F/51 y | M/ 8 m | M/5 y | M/14 y | M/16 y | F/21 y | F/21 y | F/19 y |
| **CARDIOVASCULAR** | | | | | | | | | | | | |
| Dyspnea/cyanosis | **+** | **+** | **+** | **+** | **+** | **+** | **+** | **+** | **+** | **+** | **+** | **+** |
| Arterial tortuosity | **PA, brv** | **PA, brv, aar, asa** | **PA, tha** | **PA, sav, aar** | **PA, car, aba, tha, aar** | **PA, aar, sav** | **PA, aar,**  **sav, ila** | **PA, car, tha** | **PA, car, aba, sva** | **PA, car, aba, sva** | **aar,**  **car, sva** | **aar, sva** |
| Arterial elongation | **-** | **-** | **-** | **-** | **+** |  |  |  | **-** | **+** | **+** | **+** |
| PAS | **+** | **+** | **+** | **+** | **-** | **+** | **+** | **+** | **-** | **-** | **+** | **+** |
| RV hypertension | **+** | **-** | **+** | **+** | **+** | **+** | **+** | **-** | **-** | **-** | **+** | **+** |
| RV anomalies | **+** | **+** | **+** | **+** | **-** | **-** |  | **-** | **+** | **+** | **+** | **+** |
| Aortic aneurysm | **-** | **-** | **-** | **-** | **+** | **-** | **-** | **-** | **+** | **+** | **-** | **-** |
| Arterial dissection | **-** | **-** | **-** | **-** | **-** | **-** | **-** | **-** | **-** | **-** | **-** | **-** |
| Surgery | **+** | **-** | **-** | **+** | **-** | **-** | **+** | **-** | **+** | **-** | **-** | **-** |
| Valvular regurgitation | **+** | **+** | **+** | **+** | **+** |  |  | **-** | **-** | **+** | **+** | **+** |
| **OTHER** | | | | | | | | | | | | |
| Easy bruising | **+** | **+** | **+** |  | **+** |  |  | **+** | **+** | **+** | **+** | **+** |
| Pyloric stenosis | **+** | **-** | **-** | **-** | **-** | **-** |  | **-** | **+** | **-** |  |  |
| Recurrent bronchitis | **-** | **-** | **-** |  | **-** | **+** | **-** | **-** | **+** | **+** | **+** | **+** |
| Asthenia | **+** | **+** | **+** |  | **+** |  | **+** |  | **-** | **-** | **-** | **-** |
| Diaphragm relaxation | **-** | **-** | **-** | **-** | **-** | **-** | **-** | **+** | **-** | **-** | **-** | **-** |
| Visceral prolapses | **-** | **-** | **-** | **-** | **+** | **-** | **-** | **-** | **-** | **-** | **-** | **-** |
| Chiari malformation | **-** | **-** | **-** | **-** | **-** | **-** | **-** | **+** | **-** | **-** | **-** | **-** |
| Obesity | **-** | **-** | **-** | **-** | **-** | **-** | **-** | **+** | **-** | **+** | **-** | **-** |
| Hypotonia | **-** | **-** | **-** | **-** | **+** | **-** | **-** | **+** | **-** | **-** | **-** | **-** |
| Acrogeria | **-** | **-** | **-** |  | **+** | **-** | **-** | **-** | **-** | **-** | **-** | **-** |
| Dolichocolon | **-** | **-** | **-** | **-** | **-** | **-** | **-** | **-** | **-** | **+** | **-** | **-** |
| Hypothyroidism | **-** | **-** | **-** | **-** | **-** | **-** | **-** | **-** | **+** | **+** | **-** | **-** |

*Present study, ^1^Castori et al., [9], ^2^Pilati et al., [31], ^3^Ritelli et al., [6], ^4^Vicchio et al., [12], ^5^Franceschini et al., [10], ^6^Drera et al., [8], ^7^Gardella et al., [2]; ^8^Coucke et al., [4]; ^9^Bottio et al., [11]; y: years, m: months; Hernia(s): i: inguinal, u: umbilical, l: lumbar, s: scrotal; Arterial tortuosity: PA: pulmonary arteries, aar: aortic arch, aba: abdominal aorta, asa: ascending aorta, brv: brachiocephalic vessels, car: carotid artery, ila: iliac aorta, sav: supra-aortic vessels, sva: subclavian vertebral artery, tha: thoracic aorta; PAS: stenosis of the pulmonary arteries, RV: right ventriculum.
